# Renewing Interest in Zeolites as Adsorbents for Capture of Cationic Dyes from Aqueous and Ethanolic Solutions: A Simulation-Based Insight into the Efficiency of Dye Adsorption in View of Wastewater Treatment and Valorization of Post-Sorption Materials

**DOI:** 10.3390/molecules29132952

**Published:** 2024-06-21

**Authors:** Lotfi Boudjema, Marwa Assaf, Fabrice Salles, Pierre-Marie Gassin, Gaelle Martin-Gassin, Jerzy Zajac

**Affiliations:** ICGM, Univ Montpellier, CNRS, ENSCM, Montpellier, France; lotfi.boudjema@gmail.com (L.B.); marwa.assaf.95@gmail.com (M.A.); pierre-marie.gassin@enscm.fr (P.-M.G.); gaelle.gassin@umontpellier.fr (G.M.-G.)

**Keywords:** dye adsorption, Y type Faujasite, saturation capacity, Monte Carlo simulations, solvent effect, water, ethanol

## Abstract

The impact of solvents on the efficiency of cationic dye adsorption from a solution onto protonated Faujasite-type zeolite (FAU-Y) was investigated in the prospect of supporting potential applications in wastewater treatment or in the preparation of building blocks for optical devices. The adsorption isotherms were experimentally determined for methylene blue (MB) and auramine O (AO) from single-component solutions in water and in ethanol. The limiting dye uptake (saturation capacity) was evaluated for each adsorption system, and it decreased in the order of MB–water > AO–water > AO–ethanol > MB–ethanol. The mutual distances and orientations of the adsorbed dye species, and their interactions with the oxygen sites of the FAU-Y framework, with the solvent molecules, and among themselves were inferred from Monte Carlo simulations and subsequently utilized to rationalize the observed differences in the saturation capacity. The dye–solvent competition and the propensity of the dyes to form compact pi-stacked dimers were shown to play an important role in establishing a non-uniform distribution of the adsorbed species throughout the porous space. The two effects appeared particularly strong in the case of the MB–water system. The necessity of including solvent effects in modeling studies is emphasized.

## 1. Introduction

Dyes represent common industrial environmental pollutants [1], necessitating extensive research and technological efforts to remove them from wastewater [2]. Their high solubility in water poses challenges for conventional removal methods, prompting exploration into sorption by highly porous solids as a promising alternative due to its high efficiency, ease of operation, and regeneration [3,4,5]. Simultaneously, a continuous trend towards environmentally friendly technologies imposes innovations and changes in fiber dyeing processes and this may modify the environmental issues. As an example, the fact that numerous coloring products are also soluble in ethanolic solutions can be exploited to decrease the ecological impact of conventional cotton dyeing which relies on high salt concentrations and consumes large amounts of water. Therefore, it has been proposed to carry out the dyeing process in ethanol–water mixtures containing a large excess of alcohol [6]. It is also worthwhile noting that the enhanced dye–dye aggregation behavior in concentrated aqueous solutions may be greatly reduced by adding alcohol to such an aqueous solution [7]. This highlights the importance of solvent effects in adsorbent removal capacity, thus underscoring the priority of targeted research in sorption studies.

The versatility of zeolite frameworks possessing large internal surface areas and characterized by adjustable polarity, good stability against organic solvents, acids, and bases, or high resistance to temperature and radiation makes them still interesting as potential adsorbents for removal purposes [8,9]. There has been a renewed interest in the use of zeolites with various pollutants in wastewater effluents and increased emphasis is placed on a wider comprehension of the adsorption mechanism at a molecular level [10,11,12,13,14,15,16].

In the field of Environmental Remediation, zeolitic adsorbents with a negative surface charge dominated by the permanent charge of their framework can be used to remove cationic dyes [17,18,19]. Furthermore, the advantages of zeolite hosts associated with their optical transparency in the visible region and fastness towards ultraviolet radiation may be exploited by the photocatalytic removal of dyes [20,21]. Though the role of the adsorption process in photocatalytic decomposition still remains understudied, there are some indications that the enhanced adsorption of organic pollutants in terms of adsorption capacity and kinetics has a positive impact on photocatalytic activity [21,22,23,24,25].

In a search for efficient adsorbents to be employed in Environmental Remediation, attention should also be paid to the valorization of post-sorption materials in line with the goals of Sustainable Development [5,26]. This is particularly important if the adsorbents cannot be easily regenerated and reused in successive removal cycles or if the pollutant-loaded adsorbents are of interest for the development of other processes. Chromophore-bearing zeolite systems may offer new perspectives in the preparation of building blocks for optical, electro-optical, and sensing devices (e.g., artificial photosynthesis systems, optical switches or storages, micrometer-sized lasers, and optical sensors) [27,28,29,30,31,32]. Indeed, geometrical constraints imposed by the host structure facilitate the stabilization of the optically active guest molecules in highly organized arrangements on well-defined crystallographic sites [27,30].

In the context of the above application domains, the efficiency of dye adsorption in terms of occupied surface sites (i.e., limiting dye uptake), as determined by the plateau of the individual adsorption isotherms, together with the distribution of the adsorbed species throughout the porous structure constitute the key factors to be considered in experimental and modeling studies [4,33]. Surprisingly, the role of the solvent is rarely taken into consideration [34]. One of the consequences is that the competitive mechanism of adsorption from solution is not correctly described [35,36,37].

The present paper potentially fills the gap since it examines the role of solvent in modulating the efficiency of cationic dye adsorption onto a negatively charged framework of protonated Faujasite-type zeolite (FAU-Y). Methylene blue and auramine O, chosen here as dye solutes, can be considered as representative of low-cost industrial products used in numerous coloring processes in paper, textiles, and leather industries, and also as biological stains and indicators in medicine and pharmacy. The choice of water and ethanol as solvents has been motivated by the dye solubility to yield cationic dye moieties while controlling the formation of face-to-face dimers stable in bulk solutions [38,39,40]. Therefore, the findings may potentially help validate the proposed innovations in the dyeing process carried out by making use of ethanolic solutions. As far as the preparation of chromophore-bearing zeolite materials is concerned, the goal is to track the impact of the host-guest interactions on the aggregation between two or more dye units within the zeolite cages and channels, as a function of the structure of the dye and the nature of the solvent employed. The polarity of solvent used in the preparation of dye-zeolite structures by sorption from solution should also have a significant impact, at least in relation to the surface wettability chemistry, as has been demonstrated in the case of natural sensitizing dyes anchored on the surface of mesoporous TiO_2_ [41].

In the present study, the principal molecular interactions responsible for the retention of the dye units on the zeolite surface and their distribution within the zeolite pore space were inferred from Monte Carlo simulations. The choice of the types of interacting species and their respective quantities in the zeolite structure to construct the realistic model for the adsorption system was guided by the plateau values on the experimental adsorption isotherms (i.e., saturation capacity). Inspiration in this regard was taken from selected modeling studies of gas phase adsorption relying on the adequate combination of molecular simulations and direct adsorption measurements [42,43,44,45,46]. In the present work, the mutual distances and orientations of the adsorbed dye species, their interactions with the functional groups at the adsorbent surface, with the solvent molecules, and among themselves were thoroughly described.

## 2. Results

### 2.1. Experimental Adsorption Isotherms

**Figure 1:**
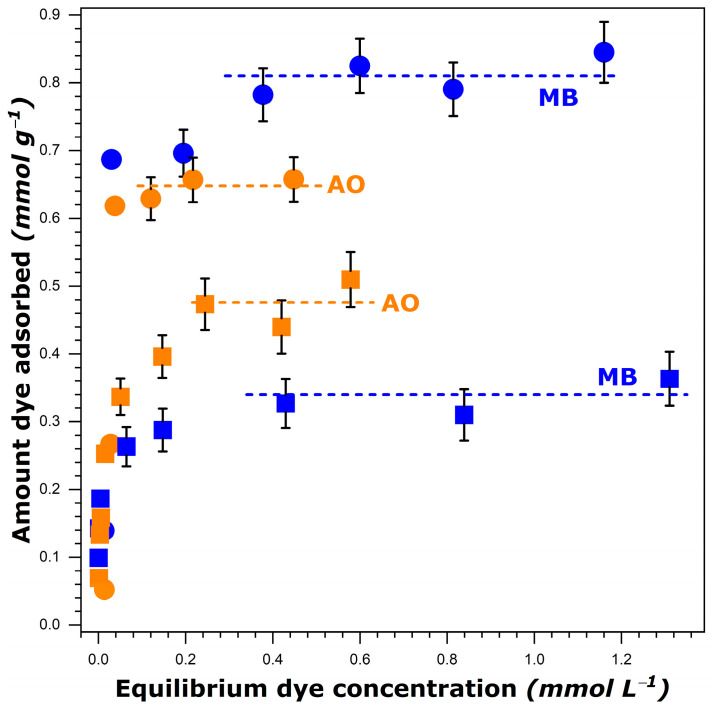
Experimental adsorption isotherms for methylene blue, MB, and auramine O, AO, dissolved in water (circles) and ethanol (squares) onto FAU-Y measured at 303 K. Error bars indicate the maximum uncertainty in the adsorption measurements (for the experimental data out of the initial quasi-vertical portion). The dashed lines represent the fitted plateau values.

The adsorption isotherms for methylene blue (further referred to as MB) and auramine O (further referred to as AO) from aqueous and ethanolic solutions at 303 K, covering the whole concentration range studied, are shown in Figure 1. They all possessed a limiting dye uptake (plateau) at higher equilibrium concentrations.

The shape of the adsorption curves reflects the fundamental difficulty in measuring dye adsorption in such systems. The amount of a given dye adsorbed per unit mass of FAU-Y is calculated according to the following equation:

(1) $$n_{a}=\frac{\left(C_{0}-C_{e}\right)\cdot V_{0}}{m_{S}}$$

where $m_{S}$ represents the mass of FAU-Y (in grams), $V_{0}$ is the initial volume of dye solution (in liters), $C_{0}$ is the initial concentration, and $C_{e}$ is the final (after attaining the adsorption equilibrium) concentration of the dye solution (in mol L^−1^). Since a sharp increase in the amount of dye adsorbed, $n_{a}$, was observed at very low equilibrium concentrations, the initial portion of the isotherm was almost vertical. Here, the difference between the initial, $C_{0}$, and equilibrium, $C_{e}$, concentrations was so small that it could not be determined with very high precision. In consequence, the uncertainty in the determination of $n_{a}$ from Equation (1) must be great in this range. For this reason, the bar errors are reported in Figure 1 only for the experimental points located out of the initial quasi-vertical portion. It should be noted that the methodology relying on the use of two different methods to analyze the dye concentrations in various ranges has been ruled out on the basis of the research experience of the research team. Moreover, the main focus of the present study is on the dye adsorption at saturation (i.e., $n_{a}^{max}$ values are of interest).

It is interesting to note that more dye species were retained by FAU-Y from aqueous solutions compared to those in ethanol, as it has been previously reported for another type of dye [47]. This decrease was particularly great in the case of MB. A simple comparison of the dye solubility in both solvents failed to adequately elucidate the observed trends. Indeed, the solubility of MB decreased only slightly when passing from water (i.e., 50.53 g L^−1^ at 303 K [40]) to ethanol (42.23 g L^−1^ at 303 K [40]), whereas the $n_{a}^{max}$ was then reduced by a factor of 2.4. Furthermore, a greater solubility of MB in comparison with that of AO in both solvents was at variance with the inversion in the adsorption capacity observed when the dye adsorption was carried out from ethanol.

It is thus impossible to rationalize the above trends on a purely experimental basis and without referring to the molecular-level description of the phenomenon.

### 2.2. Preliminary Molecular Simulations

In line with the broad principles of adsorption from solution [35,36,37], the driving force for the adsorption phenomenon usually arises as a result of the interplay between interactions operating both at the Solid–Liquid interface and within the bulk solution. Furthermore, in the case of adsorption from ionic solutions on charged solid surfaces, the phenomenon represents an ion exchange process. For example, when adsorbed from aqueous solutions, dye cations are retained in the vicinity of the negatively charged zeolite framework (i.e., pH-independent permanent charge). Therefore, the dye adsorption should follow the mechanism of cation exchange with charge-compensating protons to keep the interfacial region and the equilibrium bulk solution electrically neutral in a separate manner. The affinities of both dyes for the zeolite surface as a function of the dye’s molecular structure and size on one side, and, on the other side, the molecular interactions operating within the two solvents must be considered first.

#### 2.2.1. Model of FAU-Y

To construct the model of FAU-Y, an aluminosilicate zeolite with a general chemical formula of M_x/m_Al_x_Si_192-x_O_384_ (M—compensating cation of a charge +m, x—number of such cations in the unit cell, varying from 0 to 96) was first taken into consideration [43,48]. In the second step, the Si:Al mole ratio was fixed at 15 to correctly simulate the present experimental composition, i.e., 180 atoms of Si and 12 atoms of Al. The extra-framework H ions with a fixed charge of +1 were chosen to ensure the compensation of the global charge of the zeolite framework and to describe the exchange with dye cations (for which the global charge was also fixed at +1). The zeolite structure belonged to the *Fd3m* space group of symmetry with a unit cell of 24.85 Å [48,49]. The modification of the composition was supposed to have no effect on the unit cell parameters. The window diameter of the Faujasite supercage was equal to 1.3 nm. Figure S1 in the Supplementary Materials shows the location of the Al, Si, and O atoms in the model framework.

According to the commonly admitted view [43,48], the compensating cations were located in two crystallographic sites: site II in the supercage and sites I and I’ in the sodalite cage. Two positions for the Al substitutions, one privileging the supercage and the other the sodalite cage, were simulated to check their potential impact on the adsorption phenomenon. In both cases, the distribution of Si and Al atoms was chosen to respect Lowenstein’s distribution rule [50]. Furthermore, sodalite cages were interconnected with oxygen bridges forming a pore system with an access window having a diameter of about 0.74 nm. It appeared that the two investigated distributions had a very limited influence on the adsorption process at saturation.

#### 2.2.2. Adsorption Enthalpies and Pi-Stacking in Both Solvents

In the first step, Monte Carlo calculations were conducted by considering one dye monomer per one zeolite unit cell in view of estimating the net enthalpy of adsorption in the absence of solvent. The following values were obtained (*exothermic phenomenon*): −533.9 kJ mol^−1^, MB; −524.3 kJ mol^−1^, AO. In both cases, the interactions established between amine groups of dye monomers and oxygen centers of Faujasite were dominated by electrostatic forces and hydrogen bonds. Since these results indicate the greater affinity of MB for the zeolite surface against AO, they fail to explain the opposite trend observed when both dyes are dissolved in ethanol. An additional explanation can be searched when comparing the dye–dye interactions in pure solvents.

**Figure 2:**
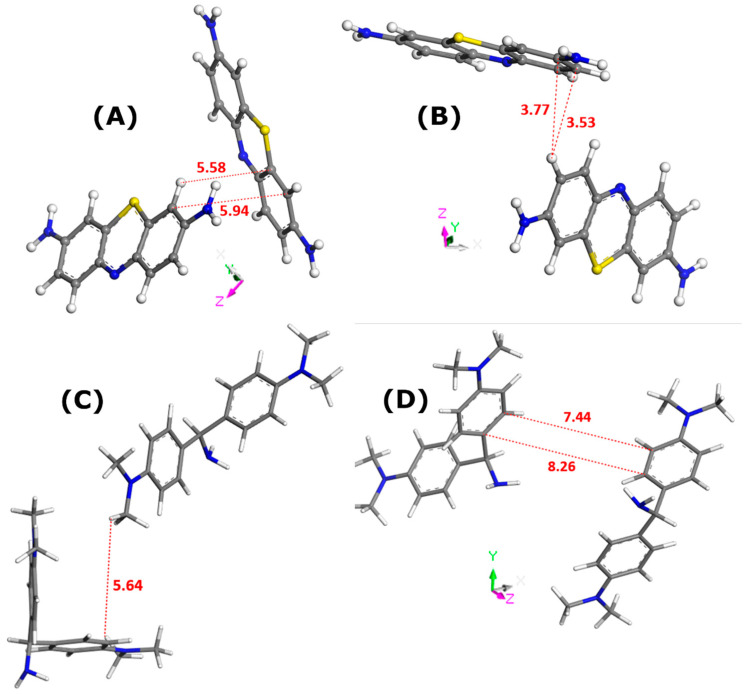
Illustration of the interactions between dye molecules in the bulk solutions, as inferred from Monte Carlo simulations; the solvent molecules have been removed for the sake of simplicity: MB in water (panel (**A**)), MB in ethanol (panel (**B**)), AO in water (panel (**C**)), and AO in ethanol (panel (**D**)). Numbers indicate the interaction distances in angstroms. The color convention for distinguishing atoms of different chemical elements was as follows: Hydrogen = white, Carbon = grey, Nitrogen = blue, and Sulphur = yellow.

Figure 2 describes the lateral interactions between dye units, as deduced from Monte Carlo calculations carried out for the bulk solutions of dyes.

In all cases, the formation of dye dimers through pi-stacking is evidenced. The two monomers in the dimer species appear to adopt orientations somewhat different from those corresponding to the sandwich-type geometry with a close approach of the dye molecules. This orientation is even almost perpendicular in the case of MB dye dissolved in water. Given the interaction distances, it is possible to predict the propensity of dye species to leave the liquid phase and to adsorb onto a solid. These interaction distances were the shortest for MB in ethanol (3.5–3.8 Å). In consequence, the MB dimers should be more stable in the ethanolic solutions. If one assumes that adsorption onto zeolite involves mostly dye monomers, it is not surprising to obtain the lowest $n_{a}^{max}$ value for the MB–zeolite–ethanol system. On the other side, even though the AO–AO distances were shorter in the case of water, thus suggesting its greater stability in the bulk solution, the maximum adsorption capacity of FAU-Y toward this dye was greater in aqueous systems.

### 2.3. Monte Carlo Simulations Involving the Presence of Solvent

It clearly follows from the preliminary study that the Monte Carlo calculations should provide the description of various molecular interactions involved in each adsorption system at the state of saturation. For this purpose, each $n_{a}^{max}$ value, as obtained from the experimental adsorption isotherms in Figure 1, was converted into the number of dye cations adsorbed per one zeolite unit cell. The remaining negative charges of the zeolite framework were compensated by protons to preserve the electroneutrality of the system. Two configurations of every adsorption system, corresponding to the same number of dye and H^+^ adsorbed species but differing in the absence or presence of the solvent were considered in the computations. Numerous trials were undertaken to determine the maximum number of solvent molecules necessary to completely fill the pore volume and obtain the overall configuration with the lowest energy.

For all systems analyzed in the present study, the dye units were found to be preferentially located within the zeolite supercages by avoiding the sodalite cages. This was obviously due to geometric constraints related to the molecular dimensions of dye cations when confronted with the size of the sodalite cage (0.74 nm) and the window diameter of the Faujasite supercage (1.3 nm). Indeed, the two dyes were considered as having a rigid structure in the Monte Carlo simulations at 303 K; this resulted in a planar MB structure with a molecular size of 1.2 nm × 0.5 nm and a bent AO geometry with a molecular projected area of 1.3 nm × 0.8 nm. Water and ethanol molecules were rather present in the entire pore space, with the only exception of hexagonal cages where the alcohol molecules could not penetrate due to their greater molecular size. The spatial distributions of various species (dye cations, protons, and solvent molecules) throughout the pore space of Faujasite were found to be heterogeneous. For example, some supercages contained one or two dye monomers, but there were no dye units in other supercages. It will be thus instructive to analyze the molecular interactions involved in dye adsorption within such supercages, as well as the estimated enthalpies of dye adsorption. The comparison with the analogous configurations where the effect of solvent has been neglected may provide arguments to illustrate the limitations of classical modeling approaches.

#### 2.3.1. MB–Zeolite System at Saturation in the Presence of Water or Ethanol

The maximum adsorption capacities of FAU-Y towards MB in the two solvents led to the following number of dye cations per unit cell: 9.4 monomers in aqueous solutions and 4.2 monomers in ethanolic solutions. To approach the real saturation state in the Monte Carlo calculations, ten MB cations and two H^+^ ions were taken in the case of water, while four MB cations and eight H^+^ ions were considered in the case of ethanol. The saturation of the pore space with the solvent was reached when, respectively, 60 molecules of water and 50 molecules of ethanol were considered to be retained within the structural unit.

**Figure 3:**
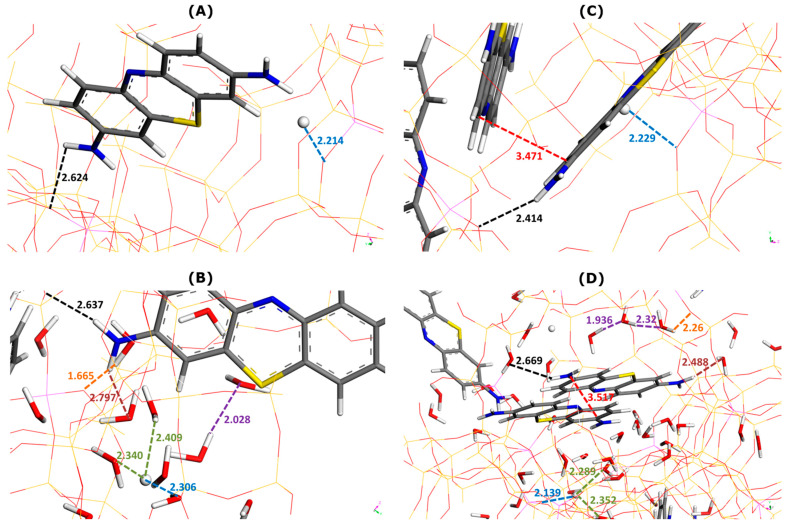
Effect of water addition on main types of interactions involved in MB adsorption onto Faujasite under saturation conditions (i.e., ten MB cations and two protons per cell), as inferred from Monte Carlo simulations. Snapshots of configurations within two selected Faujasite supercages containing one (panels (**A**,**B**)) and two MB cations (panels (**C**,**D**)). Panels B and D represent the results of simulations in which 60 water molecules have been additionally considered per cell. The colored dashed lines have been used to mark the interaction distances between various elements (numbers indicate these distances in angstroms): framework–dye (black), framework–H^+^ (blue), framework–solvent (orange), H^+^–solvent (green), dye–solvent (marron), solvent–solvent (violet), and dye–dye (red). The color convention for distinguishing atoms of different chemical elements was as follow: Hydrogen = white, Carbon = grey, Nitrogen = blue, Sulphur = yellow, and Oxygen = red.

Figure 3 illustrates the effect of water as a solvent on the main types of interactions involved in dye adsorption within two selected supercages containing one or two MB cations.

Irrespective of the presence of the solvent or not, the interactions between the adsorbed MB cations and zeolite framework appear to be chiefly of electrostatic and hydrogen-bond types involving NH_2_ groups of MB and O sites of Faujasite. The enthalpy of adsorption averaged over all adsorbed dye units decreases from −520.1 kJ mol^−1^ (without water) to −526.3 kJ mol^−1^ (with water). On average, the presence of solvent in the adsorption system favors the retention of dye cations by the zeolite (i.e., a *more exothermic phenomenon*).

It is interesting to analyze the general trends in the interaction distances for various species adsorbed within two types of supercage. When only one MB cation was located within the supercage (Figure 3A,B), the framework–dye and framework–H^+^ interaction distances slightly increased when water was included in the calculation: from 2.624 to 2.637 Å and from 2.214 to 2.306 Å, respectively.

Somewhat different trends were observed in the case of two MB cations present in the supercage (Figure 3C,D). The framework–dye distance passed from 2.414 to 2.669 Å, whereas that of the framework–H^+^ interaction decreased from 2.229 to 2.139 Å. Simultaneously, the two dye monomers interacted between themselves through pi-stacking, but the mutual orientation and interaction distance were different from those deduced from the modeling of the interactions in the bulk aqueous solution of dyes (Figure 2). Namely, the mutual alignment of the two interacting MB units corresponded more to the sandwich-type geometry with a much smaller interaction distance of 3.517 Å. Additionally, the MB monomers interacted with thirteen (Figure 3B) or nine (Figure 3D) water molecules via hydrogen bonding and the interaction distance was smaller in the case where there were two MB monomers retained within the supercage: 2.797 Å (one MB) and 2.488 Å (two MB). These trends evidence well the confinement effect in the formation of the dye dimers, which occurs within the confined pore space and is reinforced by the presence of solvent.

When there were more water molecules within the supercage (thirteen H_2_O and one MB in Figure 3B), the framework–water interaction distance was shorter (1.665 Å) than that of the water–water interaction (2.028 Å). This likely means that solvent molecules interact preferentially with the Faujasite framework. This interaction corresponded to the formation of hydrogen bonds between the hydrogen of the water molecule and the oxygen sites of the zeolite, indicating the hydrophilic character of the Faujasite sample in line with the results previously reported in the literature on X or Y-type zeolites [51,52]. Within a supercage containing two adsorbed MB cations and fewer water molecules (9 H_2_O in Figure 3D), the water–water interaction distance ranged between 1.936 and 2.32 Å, whereas that of the framework–water interaction was 2.26 Å. This result is consistent with the hypothesis that the filling of supercages with a solvent is also governed by solvent–solvent interactions to a greater extent than in the previous case (i.e., thirteen H_2_O and one MB in Figure 3B).

Some differences in the hydration of H^+^ ions can be also observed depending on the composition within the supercage: the H^+^–water interaction distance lies within the 2.340–2.409 Å range (thirteen H_2_O and one MB in Figure 3B) and within the 2.289–2.352 Å range (nine H_2_O in Figure 3D).

**Figure 4:**
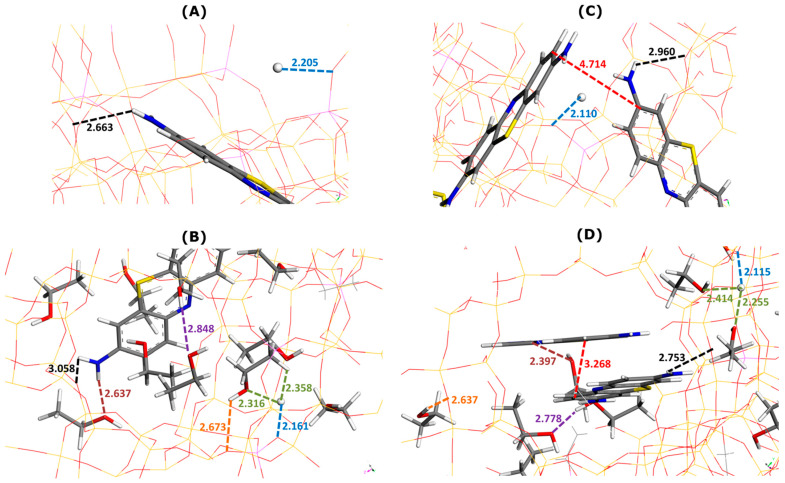
Effect of ethanol addition on main types of interactions involved in MB adsorption onto Faujasite under saturation conditions (i.e., four MB cations and eight protons per cell), as inferred from Monte Carlo simulations. Snapshots of configurations within two selected Faujasite supercages containing one (panels (**A**,**B**)) and two MB cations (panels (**C**,**D**)). Panels B and D represent the results of simulations in which 50 ethanol molecules have been additionally considered per cell. Numbers indicate the interaction distances in angstroms. The color coding used for interaction distances and atoms is the same as in Figure 3.

The interaction pattern changed to some extent in the presence of ethanol as a solvent, as can be seen in Figure 4. The NH_2_ and S groups of MB cations still interacted via electrostatic forces and hydrogen bonds with the oxygen sites of the Faujasite framework. The average enthalpy of MB adsorption increased from −482.8 kJ mol^−1^ (without ethanol) to −480.7 kJ mol^−1^ (with ethanol). The phenomenon of MB adsorption becomes much less exothermic in ethanolic media.

The framework–dye and framework–H^+^ interaction distances followed the opposite trends, depending on the composition of the supercage. In the presence of one MB cation (Figure 4A,B), the framework–dye interaction distance increased from 2.663 Å (Figure 4A) to 3.058 Å (Figure 4B) when nine ethanol molecules were additionally placed in the supercage. When two MB cations were retained inside the supercage (Figure 4C,D), this interaction distance decreased from 2.960 Å (Figure 4C) to 2.753 Å (Figure 4D) upon the addition of seven C_2_H_5_OH molecules. The framework–H^+^ interaction distance decreased from 2.205 to 2.161 Å for one MB cation (Figure 4B) and remained nearly constant at about 2.11 Å for two MB cations in the supercage (Figure 4D).

The pi-stacking of MB units was similar in terms of the mutual alignment to that observed in the pure ethanol but the interaction distance was slightly smaller (i.e., 3.268 Å in Figure 4D compared to 3.53–3.77 Å in Figure 2B). Thus, there was little impact of the confined pore space on the dye self-aggregation.

The solvation of MB cations within the supercages was due to interactions of the dye NH_2_ moieties with the OH groups of the alcohol molecules. The dye–ethanol interaction distance was equal to 2.637 Å (one MB in Figure 4B) and 2.397 Å (two MB in Figure 4D). The solvation of H^+^ ions changed when passing from the state of one MB cation (Figure 4B) to two MB cations (Figure 4D) in the supercage, but the changes were not very pronounced: the H^+^–ethanol interaction distance was within the range 2.316–2.358 Å (one MB in Figure 4B) and 2.255–2.414 Å (two MB in Figure 4D). There were also little differences in the framework–ethanol and ethanol–ethanol interaction distances between the two compositions which decreased, respectively, from 2.673 to 2.637 Å and from 2.848 to 2.778 Å.

#### 2.3.2. AO–Zeolite System at Saturation in the Presence of Water or Ethanol

The maximum adsorption capacities of FAU-Y towards AO in the two solvents corresponded to the following number of dye cations per unit cell: 7.6 monomers in aqueous solutions and 5.6 monomers in ethanolic solutions. Therefore, the saturation state in the Monte Carlo simulations was modeled by taking eight AO cations and four H^+^ ions in the case of water, whereas six AO units and six H^+^ ions in the case of ethanol. The saturation of the pore space with the solvent was attained by considering 30 molecules of water and 30 molecules of ethanol.

**Figure 5:**
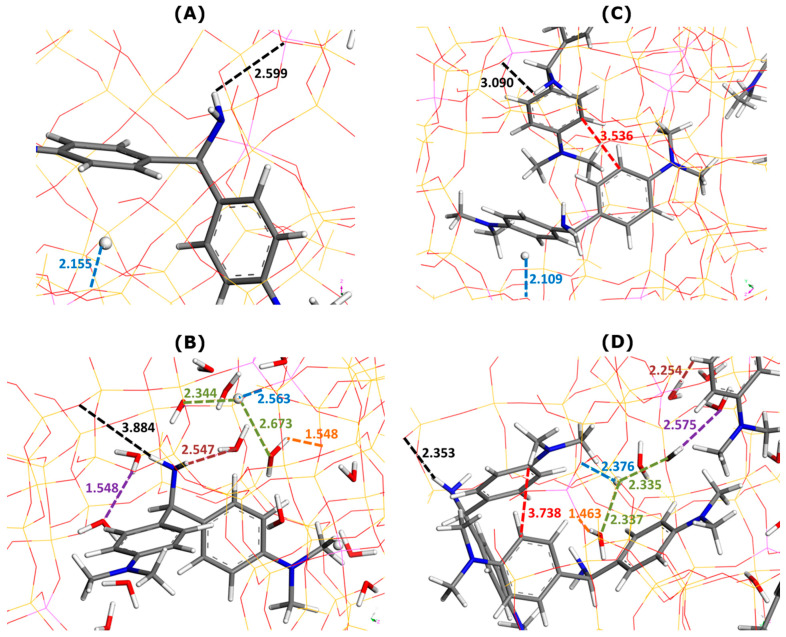
Effect of water addition on main types of interactions involved in AO adsorption onto Faujasite under saturation conditions (i.e., eight AO cations and four protons per cell), as inferred from Monte Carlo simulations. Snapshots of configurations within two selected Faujasite supercages containing one (panels (**A**,**B**)) and two AO cations (panels (**C**,**D**)). Panels (**B**,**D**) represent the results of simulations in which 30 water molecules have been additionally considered per cell. Numbers indicate the interaction distances in angstroms. The color coding used for interaction distances and atoms is the same as in Figure 3.

**Figure 6:**
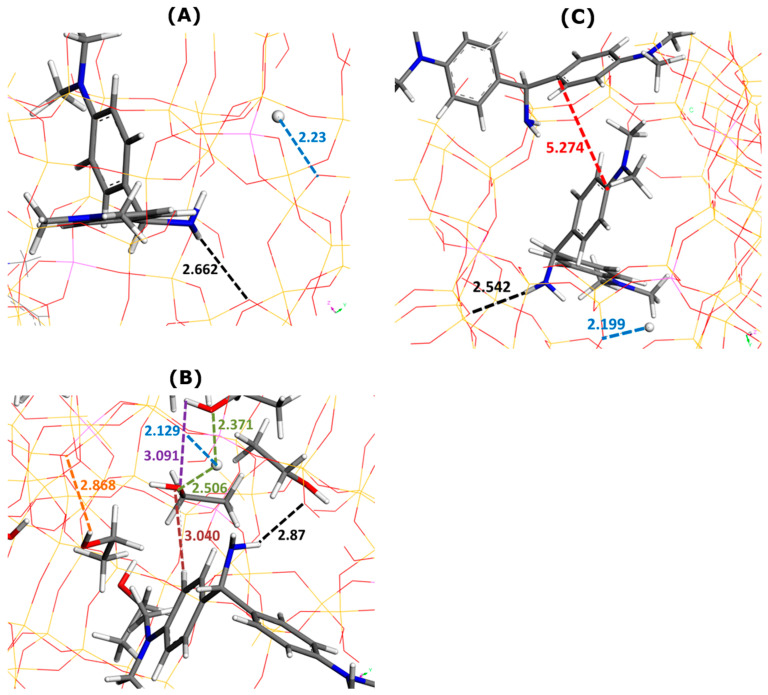
Effect of ethanol addition on main types of interactions involved in AO adsorption onto Faujasite under saturation conditions (i.e., six AO cations and six protons per cell), as inferred from Monte Carlo simulations. Snapshots of configurations within two selected Faujasite supercages containing one (panels (**A**,**B**)) and two AO cations (panel (**C**)). Panel (**B**) represents the results of simulations in which 30 ethanol molecules have been additionally considered per cell. Numbers indicate the interaction distances in angstroms. The color coding used for interaction distances and atoms is the same as in Figure 3.

Similar to the saturation state described in the previous subsection, the molecular interactions exerted by the AO cations with the zeolite framework, with solvent molecules, and between themselves are presented in Figure 5 and Figure 6. Before going into further details, it is worth noting that AO cations did not form dye dimers through π–π interactions inside the supercages when the adsorption was carried out from ethanolic solutions.

When water was used as a solvent, the enthalpy of AO adsorption increased from −525.9 kJ mol^−1^ (without water) to −514.6 kJ mol^−1^ (with water). This means that contrary to the case of MB, the presence of water in the zeolite framework makes the dye adsorption less exothermic. The interactions between the dye units and the FAU-Y framework were dominated by electrostatic forces and hydrogen bonds between AO amino groups and O sites of the Faujasite surface.

The framework–dye interaction distances were as follows: 2.599 Å (one AO without water in Figure 5A) 3.884 Å (one AO with water in Figure 5B), 3.090 Å (two AO without water in Figure 5C), and 2.353 Å (two AO with water in Figure 5D). A regular increase in the framework–H^+^ distance was evidenced when the presence of the solvent was considered in simulations: 2.155 Å (Figure 5A) vs. 2.563 Å (Figure 5B) and 2.109 Å (Figure 5C) vs. 2.376 Å (Figure 5D).

The formation of dye dimers through π–π interactions was also confirmed. Even though the mutual alignment of both monomers did not change in comparison with the configuration in bulk aqueous solutions, the dye–dye interaction distance greatly decreased from 5.64 Å (Figure 2C) to 3.536 Å (Figure 5C) or 3.738 Å (Figure 5D).

It should be also noted that ten water molecules were present in the first type of supercage, whereas only four molecules were inserted inside a supercage containing two adsorbed AO cations. The stronger hydration of dye units inside the supercage led to dye–water interaction distances shorter than those for the MB dye: 2.797 Å in Figure 3A vs. 2.547 Å in Figure 5A; 2.488 Å in Figure 3D vs. 2.254 Å in Figure 5D. The framework–water and water–water interaction distances were also shorter than the corresponding distances in the case of MB: 1.665 Å and 2.028 Å in Figure 3B vs. 1.548 Å and 1.548 Å in Figure 5B; 2.26 Å and 1.936–2.32 Å in Figure 3D vs. 1.463 Å and 2.575 in Figure 5D.

The AO monomer established interactions with both ethanol molecules and the Faujasite structure (Figure 6). Owing to its NH_2_ groups, it interacted through electrostatic forces and hydrogen bonds with the oxygen sites of FAU-Y. The average enthalpy of AO adsorption decreased from −508.4 kJ mol^−1^ (without ethanol) to −518.4 kJ mol^−1^ (with ethanol). The exothermic character of dye adsorption appeared reinforced by this solvent.

The framework–dye interaction distance increased upon solvent addition from 2.662 Å (Figure 6A) to 2.87 Å (Figure 6B). For the framework–H^+^ distance, this trend was the opposite: from 2.23 Å (Figure 6A) to 2.129 (Figure 6B). There were only five ethanol molecules in this supercage. The framework–solvent, dye–solvent, and solvent–solvent distances were, respectively, 2.868 Å, 3.040 Å, and 3.091 Å (Figure 6B). Interestingly, these values appeared much greater than those obtained for other systems. A similar conclusion may be drawn for the H^+^–ethanol distance which ranges between 2.371 Å and 2.506 Å.

When the presence of solvent was excluded in Monte Carlo simulations, the dye–dye interaction distance was predicted to be 5.274 Å (Figure 6B). The formation of AO dimers was ruled out when including ethanol in computations.

## 3. Discussion

Analyzing the experimental trends solely based on the dye affinity for the zeolite surface or on the stability of the dye units in the bulk solution was clearly insufficient to explain the differences in the maximum adsorption capacity, $n_{a}^{max}$, of FAU-Y toward MB and AO. The preferential retention of dye units within zeolitic supercages was influenced by their interactions with the aluminosilicate framework, with the solvent molecules, and also between themselves. Therefore, it is crucial to include the effect of solvent in Monte Carlo simulations.

From a thermodynamic viewpoint, the adsorption of dye monomers appeared less exothermic in the presence of solvent as the averaged enthalpies of dye adsorption were less negative than those calculated for the first adsorbed dye units without solvent (see Section 2.2.2). The exothermicity of dye adsorption (as indicated by the absolute values of adsorption enthalpy expressed in kJ mol^−1^ in parentheses), changed in the order MB–water (526.3) > AO–ethanol (518.4) ~ AO–water (514.6) > MB–ethanol (480.7). This trend did not completely follow the order of decreasing $n_{a}^{max}$ (see Section 2.1). First and foremost, it is important to realize that the averaged enthalpy value included at least two unequal contributions coming from dye units adsorbed alone in the supercage or forming a pi-stacking dimer with another adsorbed unit (note different framework–dye distances in both configurations).

According to the results reported in the previous subsections, the dye monomers were predicted to be located at greater distances from the zeolite surface when including the presence of solvent in computations. Simultaneously, the framework–solvent distances were much shorter, with the sole exception of the AO–ethanol system where the distances of both types were comparable. These arguments point towards the competition between solvent molecules and dye monomers for the oxygen sites of the zeolite framework. This competition appeared much stronger on behalf of water as a solvent since the differences between framework–dye and framework–solvent distances were greater in aqueous systems. Compared to the ethanolic systems, there were also more water molecules within such supercages and the water–water distances were generally shorter.

Furthermore, the upward trends in the zeolite–H^+^ distance were observed for aqueous systems, whereas these distances appeared to diminish when ethanol was considered as a solvent. Nevertheless, the differences between the two configurations (i.e., without and with solvent) were rather modest. This means that the competition between H^+^ and dye cations also contributes, albeit to a lesser extent, to the total competition scheme.

Less regular trends were inferred for the case where two dye monomers were retained within the supercage. This is also related to the changes in the interaction distance between two dye monomers within the supercage. Since this interaction was dominated by pi-stacking and, in all cases, the π–π interaction distance decreased compared to that reported for the bulk solution, it is reasonable to conclude that the adsorption within a confined pore space simultaneously containing molecules of a solvent favors the formation of dimers in a more compact conformation. Simultaneously, it should be more difficult for the bulkier AO units to form compact dimers within the supercages, especially when they enter into competition with a greater number of solvent molecules. In the case of the AO–ethanol system, the framework–solvent, dye–solvent, solvent–solvent, and H^+^–solvent distances were the longest among those obtained for the other systems. This likely provides an explanation as to why the formation of dimers within the supercages of FAU-Y is precluded here. As a consequence, the value of $n_{a}^{max}$ for this system was smaller than that obtained for the AO–water one, in spite of the more exothermic dye adsorption from ethanolic solutions.

## 4. Materials and Methods

### 4.1. Materials

Zeolite FAU-Y powder (Si:Al mole ratio of 15) was purchased from Zeolyst International (Conshohocken, PA, USA). Methylene blue (C_16_H_18_ClN_3_S, 97% purity, molecular weight of 319.9 g mol^−1^), auramine O (C_17_H_22_ClN_3_, 85% purity, molecular weight of 303.8 g mol^−1^), and absolute ethanol were Sigma-Aldrich products (Saint-Quentin-Fallavier, France). They all were used without further purification. Ultrapure water with a resistivity of 18.2 MΩ cm (PURELAB^®^ Chorus 1, ELGA Veolia, High Wycombe, UK) was employed to prepare aqueous solutions of both dyes.

### 4.2. Dye Adsorption Isotherms

The adsorption isotherms for both dyes from aqueous and ethanolic solutions at 303 K were determined using the solution depletion method (*c.f.*, Equation (1)). For this purpose, the appropriate dye solutions were prepared in the concentration range between 1.5 × 10^−5^ and 1.5 × 10^−3^ or 0.6 × 10^−3^ mol L^−1^ for MB and AO, respectively. The stock solutions were made by dissolving the required amount of the dye solute either in deionized water or in absolute ethanol. It is worth mentioning that the chosen dye concentrations were selected to work below the limit of dye solubility in each solvent first and then to reach a state of saturation in dye adsorption. The solubility of MB in both solvents was found to be greater than that of AO in both solvents [39,40]: 0.11 mol L^−1^ (35.84 g L^−1^) and 0.03 mol L^−1^ (~10 g L^−1^), respectively, for MB and AO in water at 293 K; 0.10 mol L^−1^ (32.19 g L^−1^) and 0.06 mol L^−1^ (~20 g L^−1^) respectively, for MB and AO in ethanol at 293 K. To attain a dye saturation plateau, it was appropriate to avoid the use of too concentrated solutions favoring the extended aggregation of dye species. However, in the case of MB in water, the dye self-aggregation to form dimers was previously reported even in the concentration range between 1·10^−6^ and 4·10^−4^ mol L^−1^ [53].

The adsorption experiments were carried out under batch conditions in stoppered Nalgene™ Oak Ridge PPCO tubes (Sigma-Aldrich, Saint-Quentin-Fallavier, France). A given mass (about 10 mg) of the FAU-Y powder was poured into 20 mL of dye solution at a given initial concentration, $C_{0}$. Tubes were stirred overnight at 30 rpm using a rotary shaker placed in a thermostated box at 303 K ± 0.5 deg. Then, the supernatant solution was separated from the solid phase by centrifugation at 10,000 rpm for 1 h. The dye concentration in the equilibrium bulk solution, $C_{e}$, was quantified with the aid of a Bruker UV–Vis spectrophotometer (Bruker, Marne la Vallée, France) by monitoring the maximum absorbance at 436 or 654 nm for AO and MB in water, and at 430 or 678 nm for AO and MB in ethanol, respectively. The supernatant was not filtered because this procedure had been previously found to affect the shape of the UV–Vis spectra.

In order to achieve a well-defined plateau in each adsorption isotherm in the concentration ranges studied, the adsorption experiments were performed at least twice by adding new points to the adsorption curves obtained in previous experiments under the same experimental conditions but at different initial concentrations. The deviations observed between the subsequent adsorption curves in the adsorption plateau region were further exploited to estimate the maximum uncertainty in the adsorption measurements.

### 4.3. Strategy of Molecular Simulations

The FAU-Y zeolite was assumed to be partially ionic with atoms carrying the following partial charges: Si (+2.4), Al (+1.4), O (−1.2), and H (+1) as extra-framework cations [54]. The partial charge distributions of dyes and ethanol were calculated based on the Electrostatic potential model (ESP) available in DMol^3^ from Materials Studio package using GGA/PW91 (Biovia, Paris, France) as a functional and DNP basis set after a geometry optimization step [55]. The TIP4P-2005 model was chosen to describe the interactions of water molecules between themselves [56]. All charges are summarized in Supplementary Materials (see Figures S2–S5, and Table S1). Each atom of the adsorbate was considered to provide a certain contribution to the adsorbate-adsorbent interactions. They were described by a sum of a repulsion–dispersion 12-6 Lennard-Jones (LJ) potential and a Coulombic term as follows:

(2) $$V_{\left(r_{ij}\right)}=\frac{q_{i}q_{j}}{4\;\pi \varepsilon_{o}r_{ij}}+4\varepsilon \left[{\left(\frac{\sigma }{r_{ij}}\right)}^{12}-{\left(\frac{\sigma }{r_{ij}}\right)}^{6}\right]$$

where ε was the depth of the potential well, σ was the distance at which the interatomic potential was zero, and $r_{ij}$ was the distance separating the atoms. All applied Lennard-Jones (LJ) parameters are summarized in Table S1. They were combined following the Lorentz-Berthelot combining rules.

Computations were performed on the basis of the above-described models of molecules, ions, and zeolite framework by using the SORPTION module available in the Materials Studio package [55]. In a typical run of computations performed in the presence of solvent, the simulation box corresponded to 1 unit cell since the unit cell parameters were large enough to use an LJ cut-off equal to 12 Å [57]. The simulations were performed using 5 × 10^6^ Monte Carlo steps for both equilibration and production steps after a loading procedure fixed at 100 × 10^6^ steps to ensure that the saturation state for solvent molecules was reached. The framework and the dye monomers were kept rigid during the whole adsorption process. The Ewald summation was used for simulating the electrostatic interactions with an accuracy fixed at 0.001 kcal mol^−1^ and a buffer width at 0.5 Å. The saturation stage was attained first by fixing the number of dye species present in the zeolite in accordance with the experimentally determined maximum adsorption capacity and the solute loading was completed with solvent molecules in a way to saturate the pore volume. Note that the experimental adsorption data were obtained in liquid solutions.

Here Monte Carlo simulations led to the identification of the most plausible configurations of dye cations and solvent molecules at the Solid–Liquid interface together with the preferential adsorption sites. The computational approach was guided by the balance of interactions operating between all parts of the adsorption system: namely, the zeolite framework, extra-framework H^+^ cations, solvent molecules, and dye cations. Note that such a research strategy was successfully utilized to describe the pore saturation with water for a Metal–Organic Framework (MOF) [58].

Supplementary Monte Carlo calculations were performed in an empty cubic box, the dimensions of which were fixed at 20 Å. During calculations, 2 dye monomers, 2 Cl^−^ anions, and solvent molecules (their number was fixed at a value close to the liquid density) were used to saturate the box. Conversely, the objective here was to determine the main plausible interactions that should be observed in the liquid phase.

## 5. Conclusions

The adsorption of the two cationic dyes, methylene blue (MB) and auramine O (AO), onto protonated Faujasite-type zeolite followed the ion-exchange pathway. The dye cations displaced charge-compensating extra-framework H^+^ ions and entered into competition with the solvent molecules to be adsorbed within the zeolitic supercages. The necessity to explicitly include the solvent effect in modeling and experimental studies of the adsorption mechanism was clearly demonstrated. The dye–solvent competition and the propensity of the dyes to form compact pi-stacked dimers played an important role in establishing a non-uniform distribution of the adsorbed species throughout the porous space at the saturation plateau. The two effects appeared particularly strong in the case of the MB–water system.

The use of MB–water, AO–water, and AO–ethanol systems should be recommended in dye removal applications. The removal procedure appears to be more efficient for MB in comparison with AO in terms of the number of retained dye units. Nevertheless, the reinforced tendency to form self-aggregates through π–π stacking interactions in the supercages (i.e., MB in water or AO in ethanol) and shorter zeolite–dye distances (i.e., MB in water) may to some extent reduce the reversibility of the adsorption phenomenon, thus rendering the regeneration step more difficult to perform.

On the side of photocatalytic decomposition of dyes in aqueous media, the existence of acid–base centers in the zeolite framework hinders the electron-hole recombination and thus enhances the photodegradation efficiency [20]. Even though the study of the decomposition mechanism is out of the scope of this work, some potential advantages of the zeolite–dye–water systems may be emphasized. The retention of adsorbates within structurally defined cages and channels of uniform sizes permits the easy isolation of the adsorbed species and thus avoids the dye–dye competition issues, which reduces the catalytic activity [25]. The adsorption of water molecules on neighboring active sites within the supercages will possibly facilitate the formation and local degradation action of the reactive radical species [24,25]. Moreover, the adsorption phenomenon follows the cation-exchange pathway, and the chloride ions, acting as common radical scavengers [24], are not co-adsorbed at the Solid–Liquid interface. Among the other parameters, it seems important to investigate the effect of the formation of dye–dye dimers upon adsorption on the overall photocatalytic activity since π–π intermolecular interactions and the delocalization of π–electrons have been found to lead to enhanced photocatalytic performance under visible light [24].

In addition, two other arguments may be put forward in favor of the use of zeolites or zeolitic supports in wastewater treatment. Firstly, the choice of natural zeolites may decrease the material cost, which is an important factor to be considered in practical uses. Secondly, given the permanent charge of the zeolite structures, their adsorption performance towards cationic adsorbates is less sensitive to changes in the pH of the surrounding medium.

The formation of dye aggregates is known to induce fluorescence quenching [7,53]. In laser technology, this aggregation is responsible for the reduction in the laser output due to the absorption of radiation by non-fluorescent aggregates combined with the quenching of the monomer [53]. The stabilization of AO units at the zeolite surface in ethanolic suspensions with a density corresponding to one AO monomer per supercage may be thus of interest in view of potential applications of chromophore-bearing zeolite materials in optics. The MB–zeolite–water system cannot prove useful in this respect since the MB dimers within the supercage adopt the sandwich-type geometry with a very close dye monomer approach.

It should be realized that the present study represents a first step towards developing industrial-scale processes. Further research effort should be devoted to investigating the underlying mechanisms, especially if the goal is to carry out the adsorption process from multicomponent industrial effluents.

## Data Availability

Data are contained within the article.

## Supplementary Materials

The following supporting information can be downloaded at: https://www.mdpi.com/article/10.3390/molecules29132952/s1, Figure S1: Model of FAU-Y zeolite adopted in the present Grand Canonical Monte Carlo simulations; Figure S2: ESP charges of Methylene Blue cation, as calculated by using DMol^3^ software [55]; Figure S3: ESP charges of Auramine O cation, as calculated by using DMol^3^ software; Figure S4: TIP4P-2005 charges for water; Figure S5: ESP charges of ethanol molecule, as calculated by using DMol^3^ software; Table S1: Force Field parameters based on the Lennard-Jones model used in this work. Reference [59] is cited in the supplementary materials.
