# Supplementary material for: Renewing Interest in Zeolites as Adsorbents for Capture of Cationic Dyes from Aqueous and Ethanolic Solutions: A Simulation-Based Insight into the Efficiency of Dye Adsorption in View of Wastewater Treatment and Valorization of Post-Sorption Materials
Source: Molecules. 2024 Jun 21;29(13):2952. doi: 10.3390/molecules29132952 (PMC11243570; doi:10.3390/molecules29132952)
Supplement: Supplementary file 1 [file molecules-29-02952-s001.zip › molecules-3050345-supplementary.pdf]

SUPPLEMENTARY MATERIALS
for

Renewing Interest in Zeolites as Adsorbents for Capture of Cationic Dyes from Aqueous and Ethanolic Solutions: A Simulation-based Insight into the Efficiency of Dye Adsorption in View of Valorization of Post-sorption Materials

Lotfi Boudjema, Marwa Assaf, Fabrice Salles*, Pierre-Marie Gassin, Gaëlle Martin-Gassin, Jerzy Zajac*

ICGM, Univ. Montpellier, CNRS, ENSCM, Montpellier, France

lotfi.boudjema@gmail.com (L.B.); marwa.assaf.95@gmail.com (M.A.);
pierre-marie.gassin@enscm.fr (P-M.G.); gaelle.gassin@umontpellier.fr (G.M-G.)
* Correspondence: fabrice.salles@umontpellier.fr (F.S.); jerzy.zajac@umontpellier.fr (J.Z.)

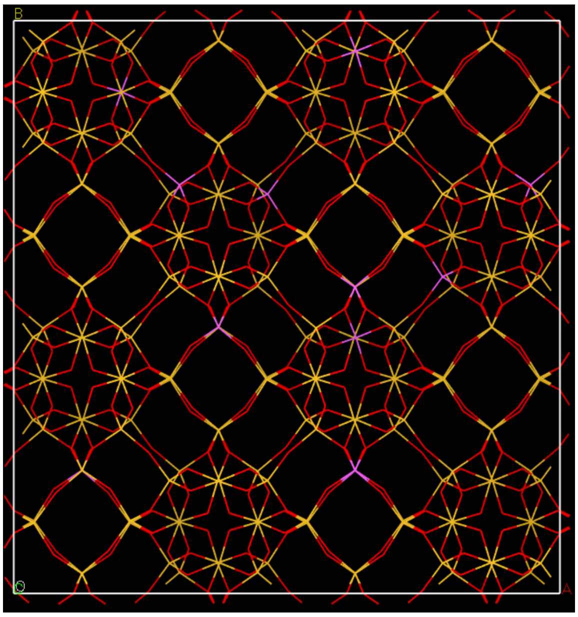

Figure S1. Model of FAU-Y zeolite adopted in the present Grand Canonical Monte Carlo simulations. The location of various atoms is indicated by making use of different colors: Al = pink, Si = yellow, O = red.

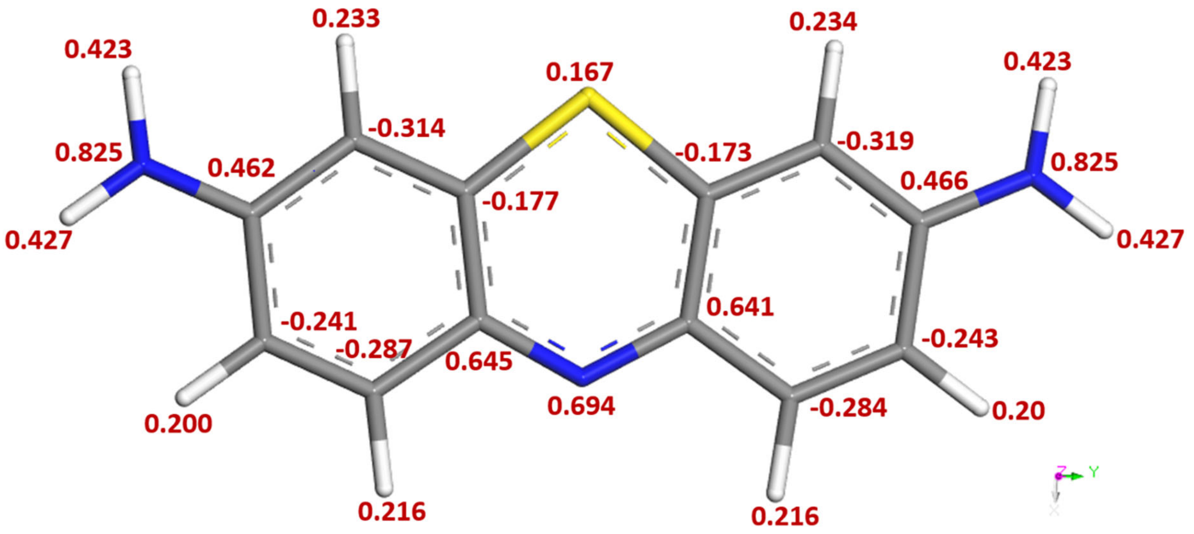

Figure S2. ElectroStatic Potential (ESP) charges of Methylene Blue (MB) cation, as calculated by using DMol[3] software. The color convention for distinguishing atoms of different chemical elements was as follow: Hydrogen = white, Carbon = grey, Nitrogen = blue, Sulphur = yellow.

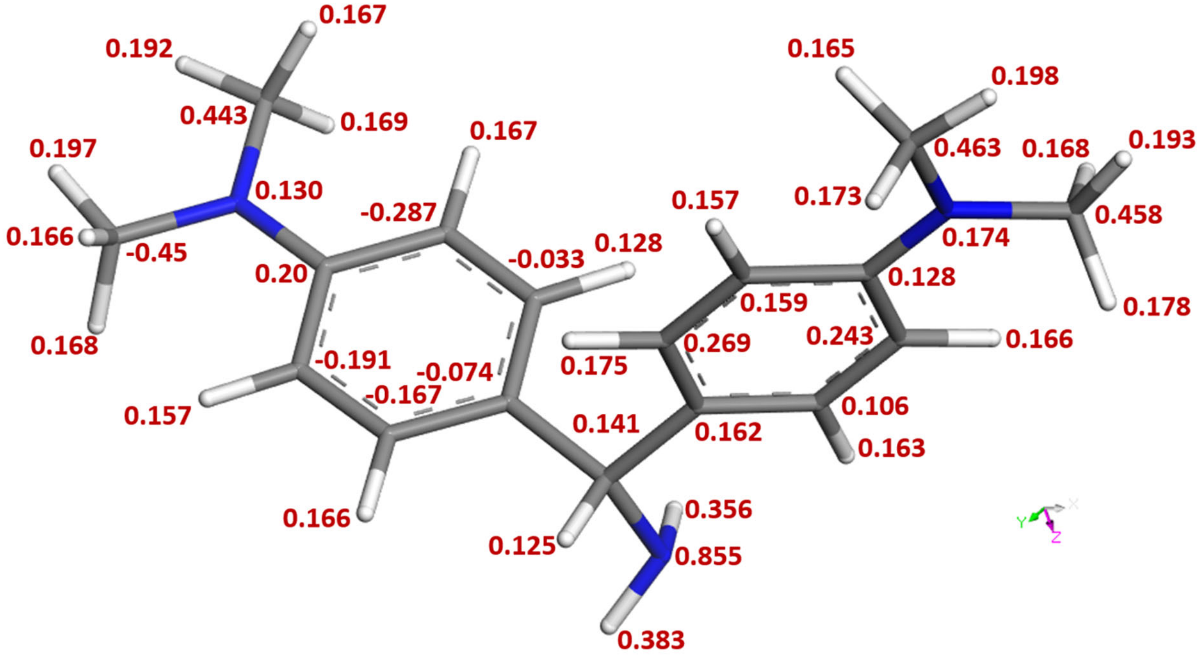

Figure S3. ElectroStatic Potential (ESP) charges of Auramine O (AO) cation, as calculated by using DMol[3] software. The color convention for distinguishing atoms of different chemical elements was as follow: Hydrogen = white, Carbon = grey, Nitrogen = blue, Sulphur = yellow.

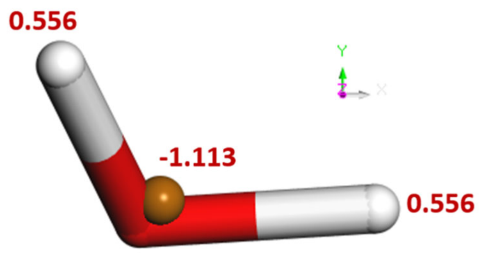

Figure S4. TIP4P-2005 charges for water. The color convention for distinguishing atoms of different chemical elements was as follow: Hydrogen = white, Oxygen = red, dummy atom = brown.

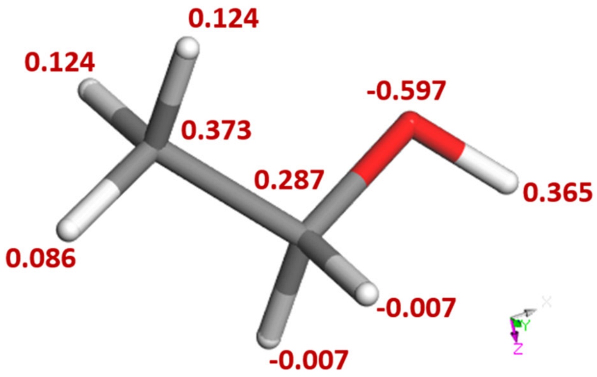

Figure S5. ElectroStatic Potential (ESP) charges of ethanol molecule, as calculated by using DMol^3 software. The color convention for distinguishing atoms of different chemical elements was as follow: Hydrogen = white, Carbon = grey, Oxygen = red.

Table S1. Force Field parameters based on the Lennard-Jones model used in this work.

	σ (Å)	ε (K)	q (|e|)
$O_{zeolithe}$	3.5	0.6	-1.2*
H^+	2.886	0.44	1.0*
$Al_{zeolithe}$	4.499	0.505	1.4*
$Si_{zeolithe}$	4.295	0.402	2.4*
H_2O	O: 3.166	O: 106.1	O: 0.0
(TIP4P-2005)	H: 0	H: 0	H: 0.5564
			M (dummy atom): -1.1128
EtOH	UFF		ESP charges**
AO	UFF		ESP charges**
MB	UFF		ESP charges**

* The charges in the zeolite structure were taken from the reference: G.J. Kramer, N.P. Farragher, B.W.H. Vanbeest, R.A. Vansanten, Phys. Rev. B, 43, 5068-5080, (1991).

** These charges are determined using DFT calculations (ElectroStatic Potential charges by $DMol^3$); they were compared with the Mulliken charges calculated by same method and a negligible influence was obtained in terms of intermolecular interactions.
